# Aspergillus nodules in chronic granulomatous disease attributable to *Aspergillus ochraceus*

**DOI:** 10.1016/j.mmcr.2017.06.004

**Published:** 2017-06-21

**Authors:** Samihah Moazam, David W. Denning

**Affiliations:** National Aspergillosis Centre, University Hospital South Manchester, Southmoor Road, Manchester M23 9LT, United Kingdom

**Keywords:** Aspergillus, Resistance, niger, Amphotericin B, Ochratoxin A

## Abstract

*Aspergillus ochraceus* is a rare pulmonary pathogen. A 39 year old male with COPD and chronic granulomatous disease presented with severe breathlessness and recurrent infections. CT scan demonstrated multiple pulmonary nodules diagnosed as chronic pulmonary aspergillosis. The patient's sputum grew *Aspergillus ochraceus* thrice over 6 months, alongside positive *Aspergillus* IgG and serum galactomannan. Despite treatment with itraconazole, the patient continued to be symptomatic. We present the first case associating *A. ochraceus* with chronic pulmonary aspergillosis.

## 1 Introduction

*Aspergillus* is a mold found throughout the environment, both in and outdoors and in the air we breathe. Several species of *Aspergillus* have been found to be pathogenic in humans, particularly in those who are immunocompromised or with underlying pulmonary conditions. The vast majority of human infections are caused by *Aspergillus fumigatus*, followed by *Aspergillus flavus* and *Aspergillus niger* [1].

Chronic pulmonary aspergillosis (CPA) is one of the conditions caused by *Aspergillus* spp. It affects individuals with underlying lung conditions, such as chronic obstructive pulmonary disease (COPD) and previous tuberculosis (TB). The disease is characterised by shortness of breath, haemoptysis, weight loss and a productive cough. Patients typically have a radiological appearance demonstrating a cavity, often containing a fungal mass, raised aspergillus IgG and positive sputum cultures and aspergillus PCR. Without antifungal therapy CPA can carry a high mortality rate over several years [2], [3], [4].

*Aspergillus ochraceus* has been rarely associated with pulmonary infections in humans. Studies and case reports have demonstrated the presence *A. ochraceus* in the context of immunocompromised and asthmatic patients, but not always as an infective agent. In a study of *Aspergillus* species found in 133 patients with suspected aspergillosis in Brazil, only 1 patient was found to have *A. ochraceus* [5]. A study in Tunisia analysed the sputum cultures from 811 neutropenic patients with haematological malignancies. From the 68 patients who did not have invasive aspergillosis, two sputum samples were positive for *A. ochraceus*, suggesting colonization rather than active infection [6]. Similarly, a study by Carpagnano et al. in 2014 demonstrated colonization with *A. ochraceus* in 3 of 43 patients with lung cancer via bronchial brushing sampling. The fungus was not found in any of the control group patients, suggesting that though an active infection was not proven, direct infection or an underlying pathogenic process contributing to lung cancer were possible [7].

*Aspergillus ochraceus* has been reported as the possible pathogen in a case of allergic bronchopulmonary aspergillosis (ABPA) reported in 1978. A 29 year old patient with a history of asthma was found to be sensitized to *A. ochraceus* and grew the species in sputum [8]. In addition, Wierzbick et al. reported a case of a 54 year old patient with invasive aspergillosis who grew *A. ochraceus* in sputum and was successfully treated with itraconazole [9].

This report describes a 39 year old male with CPA on a background of COPD and chronic granulomatous disease with growth of *A. ochraceus* in three separate sputum samples. This suggests this species was the pathogen responsible for the patient's chronic pulmonary aspergillosis.

## 2 Case

**Fig. 1:**
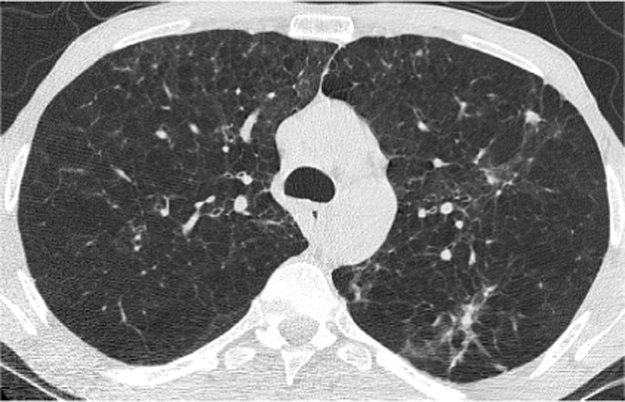
CT thorax from December 2015 demonstrating multiple pulmonary nodules.

**Fig. 2:**
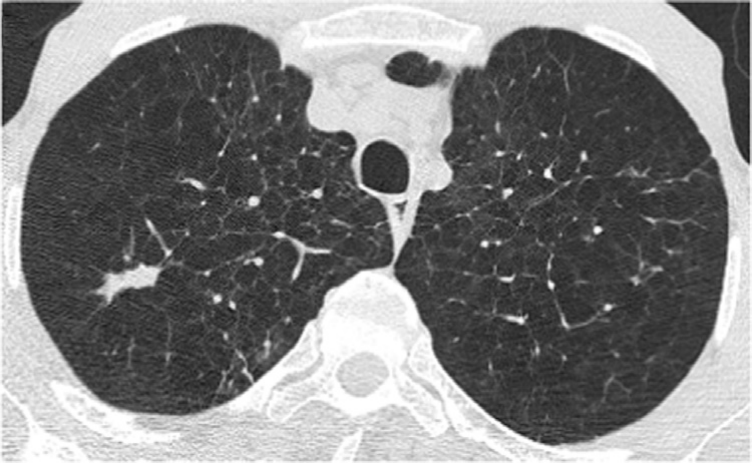
CT thorax from December 2015 showing a spiculated elongated nodule in the right upper lobe.

Patient T was 39 years old when seen in the aspergillosis clinic in December 2015 (day 0). He had been referred by the respiratory team a month earlier with symptoms of severe breathlessness with an exercise tolerance of 20 yards, recurrent infections and low oxygen saturations requiring ambulatory oxygen. He had not had any hospital admissions for the past five years. His CT scan had demonstrated multiple pulmonary nodules, including a spiculated and elongated nodule in the right upper lobe, alongside significant emphysematous changes and basal bronchiectasis (Fig. 1, Fig. 2).

Patient T had a history of COPD, diagnosed five years previously. He also had X-linked chronic granulomatous disease which had been diagnosed at the age of 4. Patient T had been admitted to ICU in February 2002 at the age of 25 with invasive aspergillosis, diagnosed from a then experimental aspergillus PCR and high aspergillus antibody levels [10]. He was treated with and responded well to IV voriconazole as an inpatient before being stepped down to oral voriconazole which he continued for 19 months. At this stage he was changed to oral itraconazole and unfortunately in 2004 was lost to follow up. Patient T was taking prophylactic co-trimoxazole, Incruse Elliptor, Relvar Ellipta, Salamol, and doxycyline PRN. He was an ex-smoker having quit 19 months previously.

Patient T was immediately started on itraconazole 200 mg BD and subsequently changed to 300 mg daily due to high levels. His aspergillus IgG was raised at 71 mg/L (< 40) (ImmunoCap, ThermoFisher) and his aspergillus precipitins (Microgen) was negative. At his next clinic appointment on day 36 his aspergillus IgG was 82 mg/L and he had a positive serum galactomannan of 1.373. His aspergillus PCR was however negative (Progenie), and his sputum showed no growth.

In April 2016, day 93, he was more breathless, had a reduced appetite with weight loss, and an increasing cough productive of green sputum. He had not had any episodes of haemoptysis or night sweats. His was *Aspergillus* IgG was now 72 mg/L, his serum galactomannan was still positive (3.103). His aspergillus PCR was again negative and one sputum sample for AAFB was also negative. His itraconazole levels at this stage were raised at > 25.6 mg/L (5–17 mg/L).

**Fig. 3:**
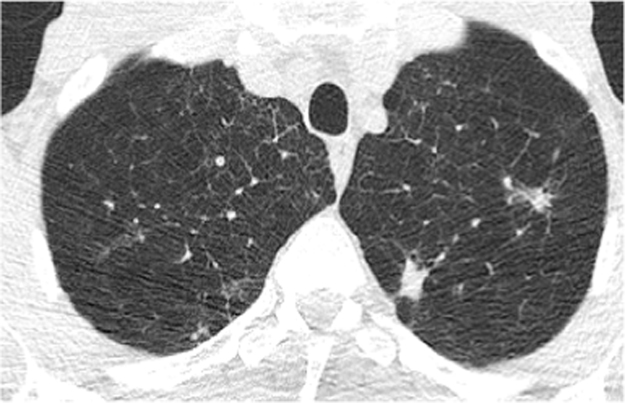
CT thorax from May 2016 demonstrating more prominent nodules.

A repeat CT scan at this stage showed significant emphysematous lung changes with more numerous bilateral and prominent nodules (Fig. 3).

On day 150 in May 2016, his sputum grew an *Aspergillus niger* in one colony, and an *A. ochraceus* in two separate samples: MIC values are in Table 1 (EUCAST method). When reviewed on day 200 he had continued to deteriorate with breathlessness and a productive cough and was now oxygen dependent. He was continuing itraconazole therapy, with good levels at 8 mg/L, and had completed two courses of oral antibiotics. When seen in clinic on day 243 he had failed to improve, though had not deteriorated further, and his *Aspergillus* IgG was 65 mg/L.

**Table 1:** MIC Values for *Aspergillus ochraceus* samples (EUCAST method).

	**April 2016**	**May 2016**	**October 2016**
**Itraconazole**	> 8	> 8	2
**Amphotericin**	8	2	> 8
**Voriconazole**	0.5	4	1
**Posaconazole**	0.25	0.5	0.5
**Isavuconazole**	0.5	8	1

By day 283 his exercise tolerance had reduced to 10–15 yards and he was started on home nebulisers by the respiratory team. His sputum again grew *A. ochraceus*, this time three colonies (Table 1). His sputum aspergillus PCR was negative, as was his serum galactomannan at 0.048.

**Fig. 4:**
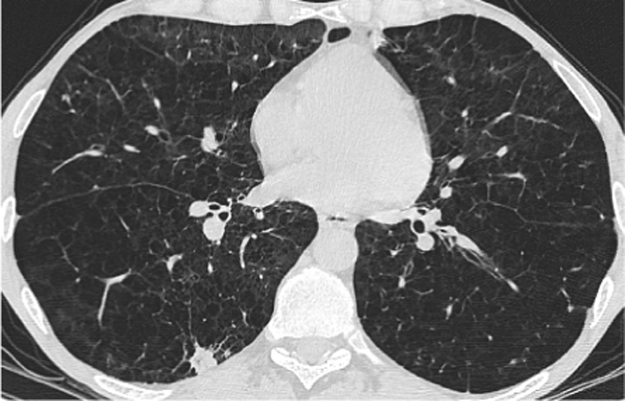
CT thorax from November 2016 showing resolution of previous inflammatory nodules.

**Fig. 5:**
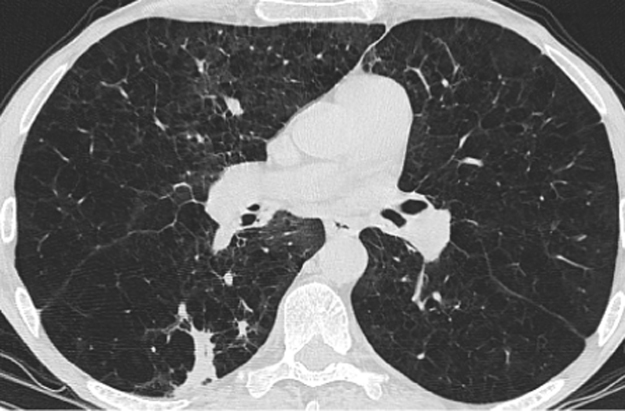
CT thorax from November 2016 showing new subpleural consolidation in right lobe.

Patient T's most recent CT scan on day 335 in November 2016 demonstrated resolution of previous inflammatory nodules but new subpleural consolidation in the right lower lobe and new consolidative foci in both upper lobes (Fig. 4, Fig. 5). The patient's most recent sputum sample grew *A. niger* from one colony, despite itraconazole levels within therapeutic range (9.4 mg/L).

## 3 Discussion

*Aspergillus ochraceus* has been rarely reported as a pulmonary pathogen in humans, and we present the first case associating this species with chronic pulmonary aspergillosis. This species appears to be itraconazole and amphotericin B resistant, although MIC breakpoints for have not been defined for *A. ochraceus.*

*Aspergillus ochraceus,* a species within the *Aspergillus* section *Circumdati,* is found throughout the environment, including as a contaminant of food products. Microscopically it appears as globose vesicles, biseriate conidiophores with ampulliform phialides. It is distinguished from other *Circumdati* species by its pink to purple brown sclerotia [11]. *A. ochraceus* is a known producer of ochratoxin A, a mycotoxin with proven pathogenic effects in animals and a possible human carcinogen [12]. Ochratoxin A has not been found in association with pulmonary disease, but is nephrotoxic in humans [13].

Chronic granulomatous disease is caused by mutations in the genes producing NADPH oxidase, resulting in recurrent infections and granulomatous lesions. Aspergillosis, including invasive disease, has been found to be associated with chronic granulomatous disease, however *A. fumigatus* and *A. nidulans* have been the most commonly found causative species [14], [15], [16]. To our knowledge, *A. ochraceus* has not been previously associated with infection in patients with chronic granulomatous disease.

There is also a question as to what extent *Aspergillus niger* has contributed to the patient's respiratory symptoms. *Aspergillus niger*, though not as common a pathogen as *fumigatus* and *flavus*, has been associated with chronic pulmonary aspergillosis, chronic necrotizing aspergillosis, and invasive aspergillosis in immunocompromised patients [17], [18], [19], [20]. The patient however has only grown *A. niger* twice, in May 2016 and November 2016, and in both cases from only one colony. This makes active infection from this species less likely, although it cannot be excluded as a second pathogen.

Interestingly, the species of *Aspergillus* causing the patient's admission in 2002 with invasive aspergillosis is unknown, although was likely to have been *A. fumigatus* as his *Aspergillus fumigatus* precipitins titre was extremely high. His *Aspergillus* PCR was positive in 2002, but his recent sputum samples have remained PCR negative and the assay cannot detect *A. ochraceus*.

There is a difficulty in assessing response to treatment for two reasons. First, the *Aspergillus* IgG assay (for *A. fumigatus)*, while elevated may not fall with therapy, as in this case. Therefore it is challenging to determine if the patient was responding to itraconazole therapy, particularly on a background of severe, deteriorating COPD. The patient continued to grow *A. ochraceus* in his sputum despite itraconazole therapy with therapeutic levels. The changing CT scan with new nodules on itraconazole is consistent with therapeutic failure, but cannot be verified with other biomarkers.

## Conflict of interest

There are none.
